# Aberrant expression of homeobox gene SIX1 in Hodgkin lymphoma

**DOI:** 10.18632/oncotarget.5556

**Published:** 2015-10-12

**Authors:** Stefan Nagel, Corinna Meyer, Maren Kaufmann, Hans G. Drexler, Roderick A.F. MacLeod

**Affiliations:** ^1^ Department of Human and Animal Cell Lines, Leibniz-Institute DSMZ – German Collection of Microorganisms and Cell Cultures, Braunschweig, Germany

**Keywords:** cooption, GRN, kernel, neural crest, NKL

## Abstract

In Hodgkin lymphoma (HL) we recently identified deregulated expression of homeobox genes MSX1 and OTX2 which are physiologically involved in development of the embryonal neural plate border region. Here, we examined in HL homeobox gene SIX1 an additional regulator of this embryonal region mediating differentiation of placodal precursors. SIX1 was aberrantly activated in 12 % of HL patient samples in silico, indicating a pathological role in a subset of this B-cell malignancy. In addition, SIX1 expression was detected in HL cell lines which were used as models to reveal upstream factors and target genes of this basic developmental regulator. We detected increased copy numbers of the SIX1 locus at chromosome 14q23 correlating with enhanced expression while chromosomal translocations were absent. Moreover, comparative expression profiling data and pertinent gene modulation experiments indicated that the WNT-signalling pathway and transcription factor MEF2C regulate SIX1 expression. Genes encoding the transcription factors GATA2, GATA3, MSX1 and SPIB – all basic lymphoid regulators - were identified as targets of SIX1 in HL. In addition, cofactors EYA1 and TLE4, respectively, contrastingly mediated activation and suppression of SIX1 target gene expression. Thus, the protein domain interfaces may represent therapeutic targets in SIX1-positive HL subsets. Collectively, our data reveal a gene regulatory network with SIX1 centrally deregulating lymphoid differentiation and support concordance of lymphopoiesis/lymphomagenesis and developmental processes in the neural plate border region.

## INTRODUCTION

Homeobox genes encode transcription factors (TFs) which regulate basic developmental processes during embryogenesis and in the adult. They share the conserved homeodomain which performs binding to both DNA and protein cofactors and serves to divide these genes into classes and subclasses [1]. Additional conserved regions found in the SINE class of homeobox genes include the SIX-domain representing an additional interface for protein interactions [2]. Homeobox genes are systematically deregulated in many types of cancer including lymphoid malignancies [3, 4]. For example, NKL subclass homeobox gene MSX1 is aberrantly activated in acute T-cell leukemia, mantle cell lymphoma, and Hodgkin lymphoma (HL) [5–7]. This gene is physiologically expressed in early lymphopoiesis and undergoes silencing upon differentiation [7, 8]. Extended activity of MSX1 or aberrant expression of other members of the NKL subclass restrains differentiation and promotes the pathogenesis of lymphoid malignancies [5, 9]. Additional homeobox genes physiologically involved in lymphopoiesis and in lymphoid malignancies if mutated or deregulated include PAX5 and ZHX2 [7, 10, 11].

HL is a B-cell malignancy in which infiltrated lymphoid tissues contain just a few malignant Hodgkin/Reed-Sternberg (HRS) cells and many bystander cells, including activated lymphocytes, plasma cells and granulocytes [12]. Deregulation of several signalling pathways mediate aberrant cell communications and promote survival [12]. Compromised B-cell development has been highlighted as a major aspect of the pathogenesis in HL from analysis of gene expression profiles of cell lines and microdissected primary HRS cells [13–15]. Main TFs important for B-cell development are absent, reduced or inactivated, including EBF1, OCT2, PAX5, TCF3, and ZHX2, generating B-cells with incomplete phenotypes. Overexpressed ID2 and ABF1 proteins and ectopic activation of the T-cell specific TF GATA3 are additional features of disturbed B-cell differentiation in HL [16, 17].

Recently, we identified aberrantly activated expression of the homeobox genes MSX1 and OTX2 in HL, mediating inhibition of B-cell differentiation [7, 18]. Interestingly, both genes are involved in the embryonal development of the neural plate border region and its descendant neural crest and placodes [19, 20]. These cells/tissues are basically involved in the development of head structures and its sensory facilities. SIX genes regulate developmental processes in this embryonic context, constituting the preplacodal region [19]. Moreover, aberrant activation of SIX1 has been reported in tumorigenesis including breast cancer, colorectal cancer and hepatocellular carcinoma, highlighting its oncogenic potential [2]. Here, we analyzed the expression, regulation and function of this homeobox gene in the context of HL, revealing an oncogenic network compromising B-cell differentiation therein.

## RESULTS

### Expression analysis of SIX1

The homeobox genes MSX1 and OTX2 are involved in the development of the neural plate border region and deregulated in HL [7, 18–20]. Homeobox genes of the SIX-family control development of the neural plate border region and its subsequent formation into placodal precursors [19, 21]. To see if members of the six strong SIX homeobox gene family also play a role in the pathogenesis of HL we first analyzed their expression in microdissected HL patient samples in silico using two different public datasets (GSE12453, Suppl. Fig. 1A and GSE39134, Suppl. Fig. 1B) [22, 23]. Both sets of these gene expression data demonstrated SIX1 and SIX3 overexpression in subsets of HL patients while SIX2, SIX4, SIX5 and SIX6 showed inconspicuous expression levels.

**Figure 1:**
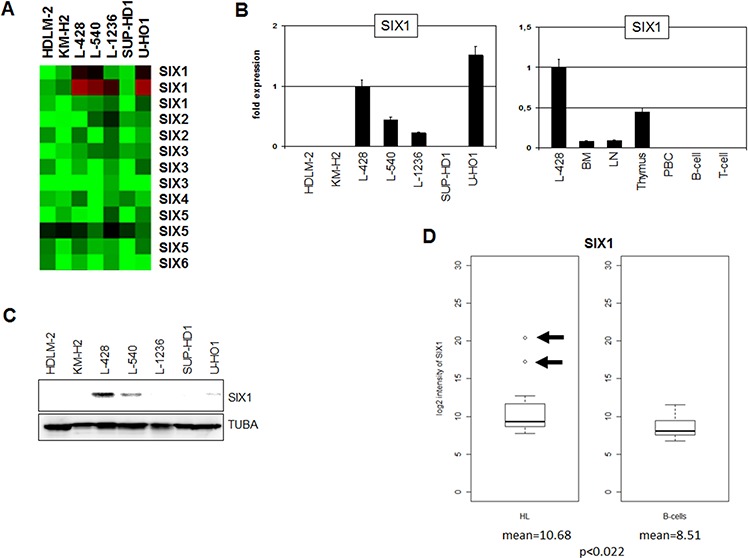
Expression of SIX1 in HL **A.** Heatmap of profiling data for SIX genes in HL cell lines demonstrates enhanced expression of SIX1 in L-428, L-540, L-1236, and U-HO1. **B.** RQ-PCR analysis of SIX1 in HL cell lines (left) and primary hematopoietic cells and tissues (right), demonstrating aberrantly enhanced SIX1 expression in L-428. **C.** Western blot analysis of HL cell lines demonstrates strong SIX1 expression in L-428 and weak expression in L-540 and U-HO1. TUBA served as loading control. **D.** In silico expression analysis of SIX1 in primary samples obtained from HL patients and from healthy donors (GSE12453). The data indicate significantly enhanced SIX1 expression levels in HL patients and show SIX1 overexpression in two patients (arrows) as compared to normal B-cells.

Expression profiling analysis of seven HL cell lines indicated overexpression exclusively of SIX1 in four cases (Fig. 1A). Quantification of SIX1 transcripts in these HL cell lines by RQ-PCR demonstrated enhanced values in L-428, L-540, L-1236 and U-HO1, supporting the profiling data and showing that L-428 and U-HO1 express the highest levels (Fig. 1B). RQ-PCR analysis of primary hematopoietic cells and tissues detected SIX1 transcripts in bone marrow (BM), lymph nodes (LN) and thymus but not in mature B- or T-cells (Fig. 1B), indicating a physiological role for this homeobox gene in early lymphopoiesis. The expression level of SIX1 in cell line L-428 was much higher as compared to the primary normal samples, implying aberrant activity in HL. Western blot analysis detected SIX1 protein in L-428, L-540 and U-HO1 but not in L-1236 (Fig. 1C). While the levels of SIX1 RNA and protein correlate in L-428, L-540 and L-1236, the discrepant expression levels in U-HO1 may suggest posttranslational regulation in this cell line.

Statistical analysis of in silico expression data for SIX1 (dataset GSE12453) indicated significantly enhanced values (*p* < 0.022) as compared to B-cells from healthy donors and demonstrated overexpression in 2/17 (12%) of HL patients (Fig. 1D). Aberrant overexpression in both patients and cell lines indicts SIX1 in the pathology of HL. This prompted further examination of the regulation and function of this homeobox gene using SIX1-positive cell lines as models.

### Genomic and promoter analyses of SIX1

**Figure 2:**
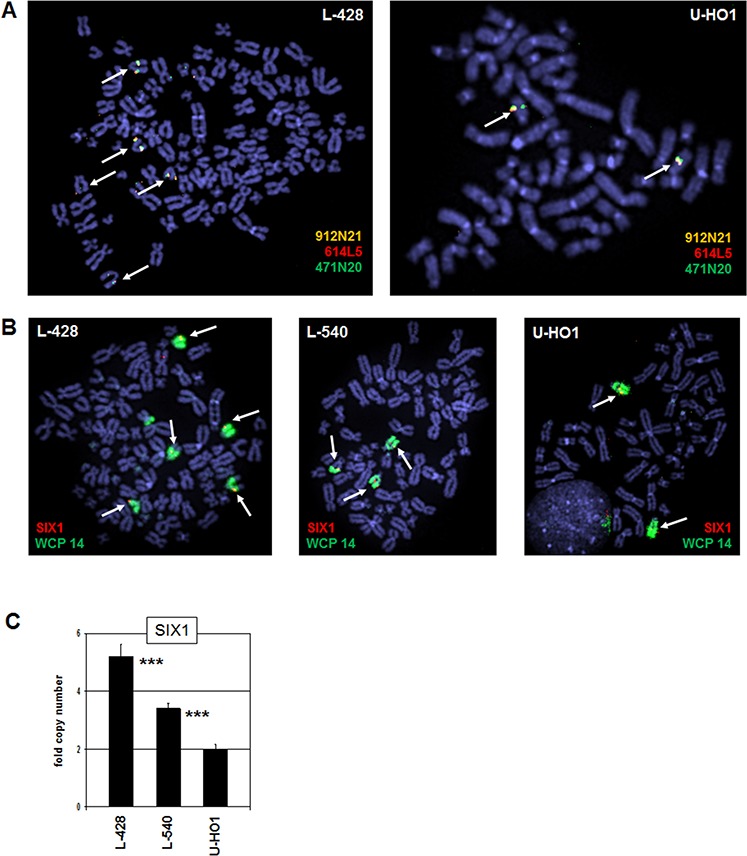
Chromosomal and genomic analysis of SIX1 **A.** FISH analysis of the SIX1 locus in L-428 (left) and U-HO1 (right) using flanking and covering probes indicates absence of chromosome 14q23 translocations. The names of used BAC clone probes and their labelled colors are indicated. Arrows highlight the SIX1 loci hybridizing all three probes. **B.** FISH analysis in L-428, L-540, and U-HO1 using a SIX1 probe in combination with a WCP probe for chromosome 14 indicates five SIX1 copies in L-428, three copies in L-540 and two copies in U-HO1. Arrows highlight the SIX1 loci. **C.** Quantification of SIX1 genes by RQ-PCR in genomic DNA of L-428, L-540, and U-HO1 confirmes the copy numbers in these cell lines as indicated by FISH analysis. The calculated *p*-values are indicated by asterisks (*p < 0.05, **p < 0.01, ***p < 0.001, n.s. no significance).

The SIX1 gene is located at chromosomal band 14q23.1. To detect potential genomic aberrations at SIX1 in HL we performed fluorescence in situ hybridization (FISH) analyses using BAC probes covering coding and flanking regions (Fig. 2A), and whole chromosome painting (WCP) probes to highlight chromosome 14 (Fig. 2B). Together, these data excluded chromosomal rearrangements at the SIX1 locus in L-428, L-540 and U-HO1 (data not shown for L-540), but demonstrated copy number gains in L-428 and L-540. Consistently, RQ-PCR analysis of genomic DNA of HL cell lines confirmed the chromosomal data for SIX1 gene copy numbers, showing two copies in U-HO1, three in L-540, and five in L-428 (Fig. 2C). Thus, we identified gains of wild type configured SIX1 loci in HL cell lines which may contribute to the aberrantly enhanced activity of this homeobox gene.

**Figure 3:**
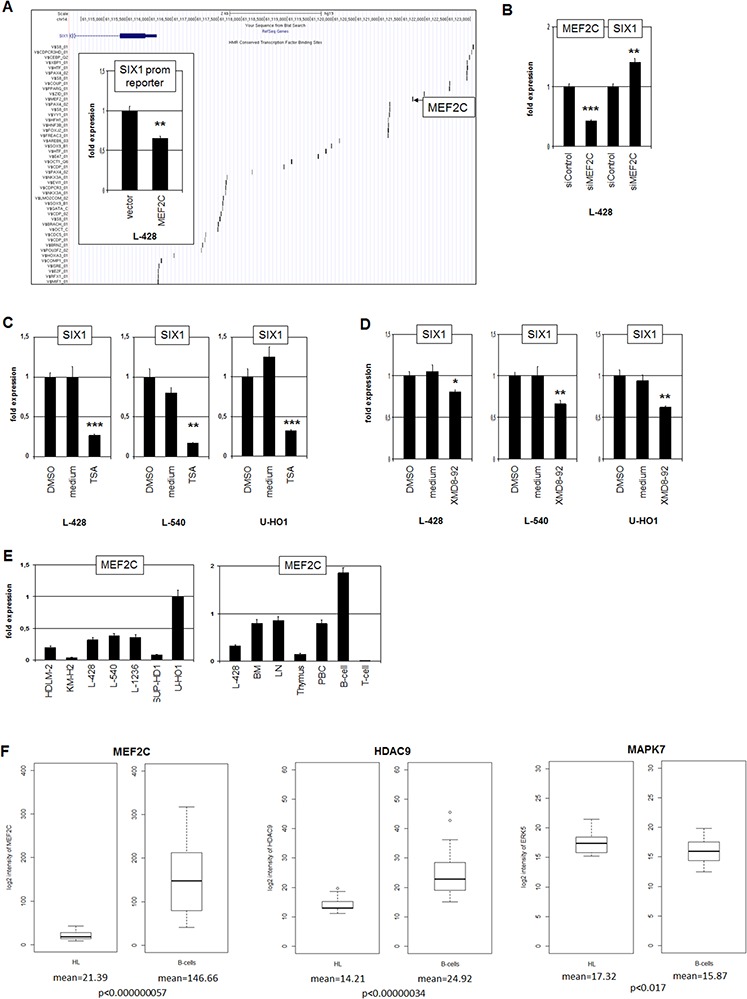
MEF2C inhibits SIX1 in HL **A.** The regulatory upstream region of SIX1 contains several potential binding sites for TFs, including MEF2C at −5593 bp (UCSC Genome Bioinformatics). Reporter gene assay analysis of this binding site in L-428 cells (insert) demonstrates an inhibitory impact of MEF2C on SIX1 transcription. **B.** RQ-PCR analysis after siRNA-mediated knockdown of MEF2C in L-428 cells resulted in increased SIX1 expression, indicating suppression of SIX1 by MEF2C. **C.** RQ-PCR analysis of SIX1 transcripts in HL cell lines after treatment with HDAC-inhibitor TSA shows decreased expression levels. **D.** RQ-PCR analysis of SIX1 transcripts after treatment of HL cell lines with MAPK7-inhibitor XMD8–92 shows decreased expression levels. **E.** RQ-PCR quantification of MEF2C transcripts in HL cell lines (left) and primary hematopoietic cells and tissues (right) demonstrates reduced expression of MEF2C in L-428 as compared to BM and B-cells. **F.** In silico expression analysis of MEF2C (left), HDAC9 (middle), and MAPK7 (right) in primary samples obtained from HL patients and from healthy donors (GSE12453). The data indicate significantly reduced expression levels of MEF2C and HDAC9 and enhanced levels of MAPK7 in HL patients as compared to normal B-cells.

To identify transcriptional regulators contributing to SIX1 deregulation in HL we analyzed the promoter region of this homeobox gene using dataset GRCh37/hg19 (www.genome-euro.ucsc.edu). This exercise revealed several potential TF binding sites including one for the B-cell specific regulator MEF2C at −5593 bp (Fig. 3A). SiRNA-mediated knockdown of MEF2C in L-428 resulted in elevated expression levels of SIX1, indicating an inhibitory impact of MEF2C on SIX1 (Fig. 3B). Analysis of the promoter section which contains the identified binding site by reporter gene assay confirmed this inhibitory role, demonstrating direct regulation of SIX1 by MEF2C (Fig. 3A). However, genomic sequence analyses of this MEF2C binding site in L-428, L-540 and U-HO1 cells indicated the absence of mutational alterations (data not shown).

The activity of MEF2C protein is regulated by posttranslational modifications including acetylation and phosphorylation. Phosphorylation by MAPK7/ERK5 supports the potential of MEF2C to activate transcription [24]. In contrast, acetylation of MEF2C enhances its inhibitory activity and deacetylation by HDAC9 reverses this effect [25, 26]. To analyze the impact of protein acetylation on SIX1 expression we treated HL cell lines L-428, L-540 and U-HO1 with HDAC-inhibitor Trichostatin A (TSA). Subsequent RQ-PCR analysis demonstrated suppression of SIX1 transcription (Fig. 3C), confirming the likely impact of HDAC activity on SIX1 expression via MEF2C. To analyze the influence of protein phosphorylation on SIX1 expression we treated HL cell lines L-428, L-540 and U-HO1 with MAPK7-inhibitor XMD8–92. RQ-PCR analysis demonstrated suppression of SIX1 transcription (Fig. 3D), supporting SIX1 regulation via phosphorylation of MEF2C by MAPK7.

The expression of MEF2C was quantified in HL cell lines by RQ-PCR, demonstrating varying transcript levels (Fig. 3E). The analysis of MEF2C expression levels in primary hematopoietic cells and tissues in comparison to HL cell line L-428 is shown in Fig. 3E. MEF2C expression increases in step with B-cell differentiation as indicated by a two-fold enhanced transcript level in B-cells as compared to BM. In T-cells no MEF2C RNA was detectable at all, supporting its B-cell specific role in lymphoid development. The level of MEF2C transcripts in L-428 is even lower as compared to BM, indicating aberrant downregulation in this B-cell malignancy. Accordingly, in silico analysis of MEF2C expression in HL patient samples relative to B-cells from healthy donors demonstrated significantly reduced levels in HL (Fig. 3F). Analysis of the same dataset for HDAC9 and MAPK7 demonstrated for HL patients significantly reduced and elevated expression levels, respectively (Fig. 3F). Thus, we observed in HL aberrantly reduced MEF2C expression and altered levels of two MEF2C-modifiers which collectively degrade the inhibitory potential of this B-cell specific TF.

### Network analysis

To identify both additional upstream factors (de)regulating SIX1 expression and downstream target genes of SIX1 in HL we performed expression profiling of SIX1-positive cell lines (L-428, L-540, U-HO1) and controls (HDLM-2, KM-H2, SUP-HD1). The comparison of both groups yielded two lists showing genes with respective elevated and reduced expression intensities of at least 4-fold (Suppl. Tables 1A, 1B). Selected candidates comprised the overexpressed genes GATA2, SIX1, and WNT3 and downregulated genes FGF2, HDAC9, SPIB, and TGFBR3. Reduced expression levels of HDAC9 in SIX1-positive cell lines correspond to MEF2C modulation supporting our data as described above. The other gene candidates were examined in more detail and are discussed below.

**Figure 4:**
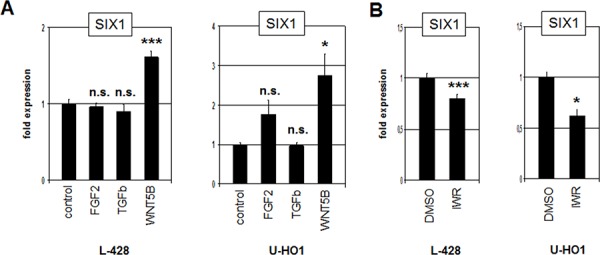
WNT-signalling activates SIX1 expression in HL **A.** RQ-PCR analysis of L-428 and U-HO1 cells after treatment with FGF2, TGFb and WNT5B demonstrates activation of SIX1 expression by the WNT-pathway. **B.** RQ-PCR analysis of L-428 and U-HO1 cells after treatment with WNT-signalling inhibitor IWR confirms activation of SIX1 expression by WNT-signalling.

Most homeobox genes are regulated during development by multiple conserved signalling pathways [27, 28]. To examine if the FGF2-, TGFb- and WNT-pathways are involved in the regulation of SIX1 expression we treated L-428 and U-HO1 cells with recombinant FGF2, TGFb and WNT5B proteins for 20 h. While the expression level of SIX1 did not change significantly after treatment with FGF2 and TGFb, SIX1 transcription increased clearly after WNT5B treatment in both cell lines (Fig. 4A). Moreover, treatment of L-428 and U-HO1 with WNT-signalling inhibitor IWR mediated reduction of SIX1 expression (Fig. 4B), supporting an activatory impact of the WNT-signalling pathway on SIX1 in HL.

### SIX1 (de)regulates lymphopoietic transcription factors

**Figure 5:**
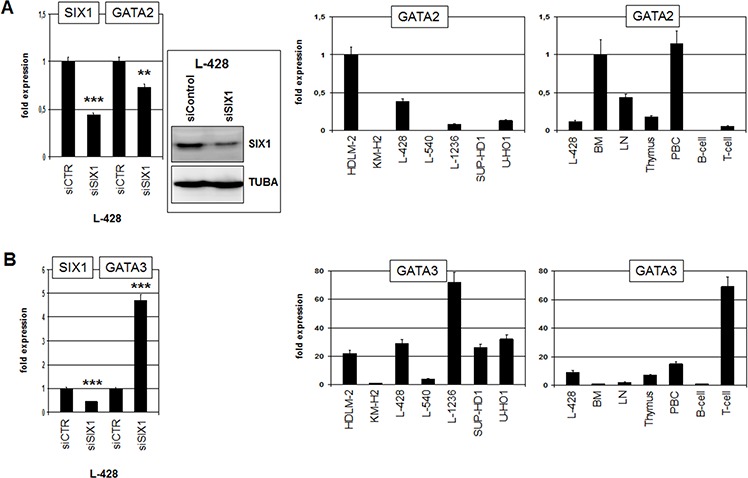
SIX1 regulates expression of GATA2 and GATA3 **A.** RQ-PCR analysis of GATA2 after siRNA-mediated knockdown of SIX1 in L-428 cells (left), demonstrating activation of GATA2 transcription by SIX1. Western blot analysis (left) confirmed reduced SIX1 expression after siRNA-mediated knockdown in L-428 cells. Quantification of GATA2 by RQ-PCR in HL cell lines (middle) and primary hematopoietic cells and tissues (right), demonstrating aberrant GATA2 expression in L-428 as compared to B-cells. **B.** RQ-PCR analysis of GATA3 after siRNA-mediated knockdown of SIX1 in L-428 cells (left), demonstrating suppression of GATA3 transcription by SIX1. Quantification of GATA3 by RQ-PCR in HL cell lines (middle) and primary hematopoietic cells and tissues (right), demonstrating GATA3 overexpression in L-428 as compared to B-cells.

Next we examined if candidate transcription factors GATA2 and SPIB are target genes of SIX1 in HL. In accordance with such a role, siRNA-mediated knockdown of SIX1 resulted in reduced expression of GATA2 (Fig. 5A), demonstrating that SIX1 activates GATA2 transcription. Expression analysis of GATA2 in HL cell lines indicated variable transcript levels with HDLM-2 and L-428 showing the highest values (Fig. 5A). However, FISH analyses in HDLM-2 excluded chromosomal aberrations at the GATA2 locus (data not shown). Expression of GATA2 in primary hematopoietic cells and tissues was high in BM and absent or low in B-/T-cells (Fig. 5A), indicating its downregulation during lymphoid development. Consistently, GATA2 is a regulator of early hematopoietic development [29]. Aberrant upregulation of GATA2 during lymphoid development may inhibit B-cell differentiation [30], representing one possible mechanism of transforming activity of SIX1 in HL.

GATA3 is an hematopoietic regulator of T-cell development and aberrantly activated in HL [17, 31]. Therefore, we analyzed GATA3 expression after knockdown of SIX1 in HL cell line L-428 as well, surprisingly showing that SIX1 mediates inhibition of GATA3 (Fig. 5B). In primary cells GATA3 was weakly expressed in BM and B-cells but highly activated in T-cells (Fig. 5B), supporting a regulatory role in T-cell differentiation. L-428 cells showed higher GATA3 levels as compared to BM and B-cells although SIX1 mediates reduction of GATA3 in this cell line. Of note, L-1236 cells express the highest level of GATA3 and SIX1 protein expression therein evidenced downregulation (see above). Furthermore, the remarkable expression of SIX1 in the thymus combined with its absence in T-cells, as shown above (Fig. 1B), suggests that the identified inhibitory role of SIX1 for GATA3 may operate physiologically in early T-cell differentiation.

**Figure 6:**
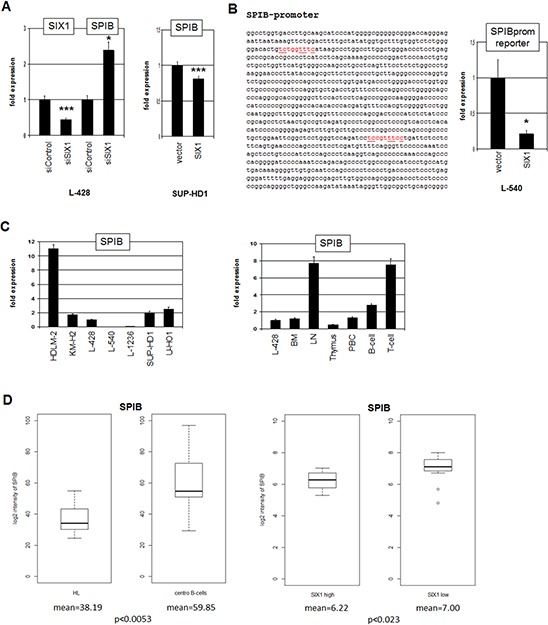
SIX1 inhibits SPIB expression in HL **A.** RQ-PCR analysis of SPIB after siRNA-mediated knockdown of SIX1 in L-428 cells (left), demonstrating suppression of SPIB transcription by SIX1. Forced expression of SIX1 in SUP-HD1 cells results in reduced expression of SPIB (right), supporting its suppressive potential. **B.** Sequence analysis of the SPIB promoter region revealed two potential SIX1 binding sites at −892 bp and −320 bp (marked in red). Reporter gene assay analysis of these binding sites demonstrates an inhibitory impact of SIX1 on SPIB transcription (right). **C.** Quantification of SPIB by RQ-PCR in HL cell lines (left) and primary hematopoietic cells and tissues (right), demonstrating reduced SPIB expression in L-428 as compared to B-cells and LN. **D.** In silico expression analysis of SPIB in primary samples obtained from HL patients and from germinal center-B-cells of healthy donors (left, GSE12453). The data indicate significantly reduced SPIB expression levels in HL patients. In silico expression analysis of SPIB in primary samples, obtained from SIX1-positive and SIX1-negative HL patients (right, GSE12453). The data indicate significantly reduced SPIB expression levels in SIX1-positive HL patients.

To determine the impact of SIX1 on SPIB we quantified SPIB transcripts after siRNA-mediated knockdown of SIX1 in L-428 (Fig. 6A), showing increased expression levels. Correspondingly, forced expression of SIX1 in (SIX1-negative) SUP-HD1 cells inhibited expression levels of SPIB (Fig. 6A). Collectively, these data show that SIX1 inhibits SPIB transcription. Sequence analysis of the promoter region of SPIB identified two potential SIX1 binding sites. Therefore, reporter gene assay of a genomic fragment comprising both sites was performed in L-540 cells (Fig. 6B). Forced expression of SIX1 reduced the reporter gene activity demonstrating that SIX1 suppresses SPIB directly.

Quantification of SPIB expression in HL cell lines demonstrated variable transcript levels with HDLM-2 showing the highest, L-540 and L-1236 the lowest values (Fig. 6C). In primary cells SPIB showed highest expression levels in LN, T- and B-cells while BM and thymus expressed lower levels, indicating that SPIB represents a late lymphoid differentiation factor (Fig. 6C). In silico expression analysis of SPIB demonstrated significantly reduced transcript levels in HL patients as compared to germinal center B-cells of healthy donors, and SIX1-positive HL patients express lower levels as compared to SIX1-negative patients (Fig. 6D). Therefore, SIX1 contributes to the observed aberrant suppression of SPIB in HL.

**Figure 7:**
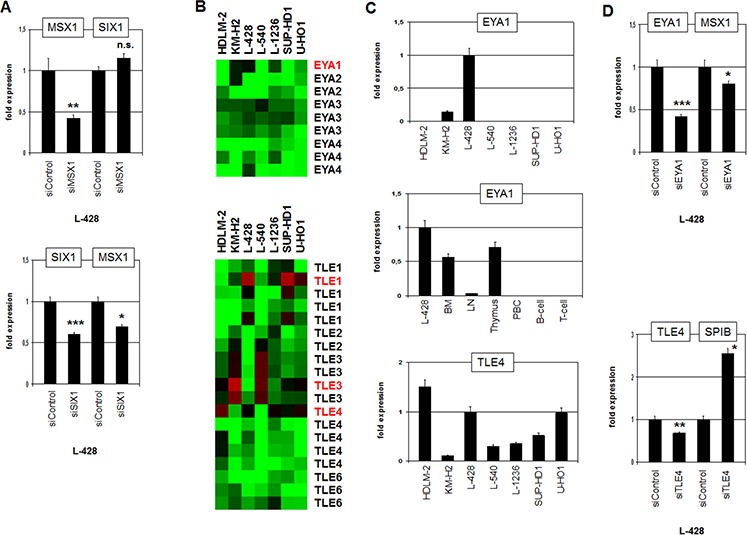
Analysis of MSX1 regulation and of SIX1-cofactors EYA1 and TLE4 **A.** RQ-PCR analysis of SIX1 after siRNA-mediated knockdown of MSX1 in L-428 cells (above) demonstrates absence of regulation. RQ-PCR analysis of MSX1 after siRNA-mediated knockdown of SIX1 in L-428 cells (below) demonstrating activation of MSX1 by SIX1. **B.** Heatmap of profiling data for EYA genes (above) and TLE genes (below) in HL cell lines demonstrates elevated expression of EYA1, TLE1 and TLE4 expression in L-428 (indicated in red letters). **C.** Quantification of EYA1 by RQ-PCR in HL cell lines (above) and primary hematopoietic cells and tissues (middle), demonstrating EYA1 overexpression in L-428. Quantification of TLE4 by RQ-PCR in HL cell lines (below) shows elevated TLE4 expression in L-428. **D.** RQ-PCR analysis of MSX1 after siRNA-mediated knockdown of EYA1 in L-428 cells (above), demonstrating activation of MSX1 transcription by SIX1-cofactor EYA1. RQ-PCR analysis of SPIB after siRNA-mediated knockdown of TLE4 in L-428 cells (below) demonstrates suppression of SPIB transcription by SIX1-cofactor TLE4.

Comparative expression profiling analysis revealed no significant difference in MSX1 expression between SIX1-positive and -negative HL cell lines (see above). Nevertheless, MSX1 represents together with SIX1 master regulators in the neural plate border region and is deregulated in HL as well [7, 19, 20]. To analyze a potential regulatory relation between SIX1 and MSX1 we performed siRNA-mediated knockdown of MSX1 in L-428. However, SIX1 expression emerged unaltered discounting SIX1 regulation by MSX1 (Fig. 7A). In contrast, siRNA-mediated knockdown of SIX1 resulted in reduced expression levels of MSX1 (Fig. 7A), demonstrating an activating impact of SIX1 on MSX1 expression and revealing MSX1 as an additional gene deregulated by SIX1 in HL.

Thus, we have identified both positive and negative target genes of homeodomain TF SIX1 in HL. In embryonal development of the placodal region SIX1 mediates target gene activation by interaction with EYA proteins and suppression by interaction with TLE proteins [32]. To examine if these physiological interactions are conserved in HL, we first analyzed which of these cofactors are present in HL cells. Expression profiling data revealed that EYA1, TLE1, TLE3 and TLE4 are well expressed in HL cell lines (Fig. 7B). RQ-PCR analyses confirmed the profiling data, showing that L-428 expresses EYA1 and TLE4 (Fig. 7C). Of note, EYA1 exhibited high expression levels in BM and thymus indicating a physiological role in early hematopoiesis and T-cell development (Fig. 7C). The expression level of EYA1 in L-428 demonstrated higher values indicating aberrant overexpression. SiRNA-mediated knockdown of EYA1 in L-428 resulted in reduced expression of MSX1 implicating EYA1 in its coactivation along with SIX1 (Fig. 7D). SiRNA-mediated knockdown of TLE4 resulted in enhanced expression of SPIB, indicating a corepressive function (Fig. 7D). Thus, our data support the conclusion that SIX1 operates in HL as activatory and inhibitory TF according to the particular interacting cofactor involved.

## DISCUSSION

**Figure 8:**
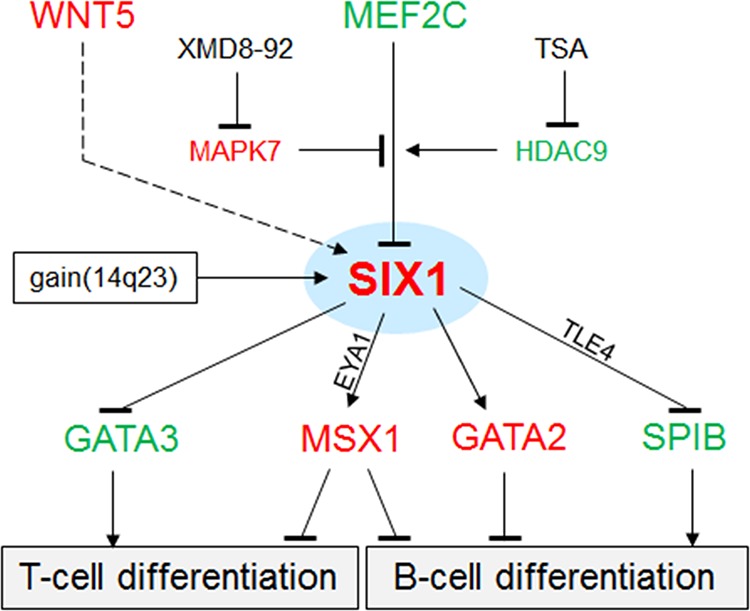
Gene regulatory network (GRN) of SIX1 in HL This GRN summarizes the results obtained in our study. SIX1 is located at a central position targeted by copy number gains. The WNT-signalling pathway (dashed line) activates SIX1 expression. MEF2C regulates SIX1 transcription depending on posttranslational modifications by MAPK7 (inhibits the suppressive function of MEF2C) and HDAC9 (activates the suppressive function of MEF2C). Target genes regulated by SIX1 include GATA3, MSX1, GATA2, and SPIB. Their deregulation may inhibit B-cell differentiation processes. EYA1 and TLE4 may operate as activatory and inhibitory cofactors, respectively. Red: elevated expression level, green: reduced expression level.

The results of this study are summarized in a gene regulatory network (GRN) as shown in Fig. 8. The deregulated homeobox gene SIX1 is located at a central position, activated by copy number gains at chromosome 14q23 and by the WNT-signalling pathway, but inhibited by the TF MEF2C. MEF2C expression is reduced in HL and its inhibitory potential diminished accordingly. Target gene regulation of SIX1 includes activation of GATA2 and MSX1 and repression of GATA3 and SPIB. Collectively, this aberrant GRN disturbed the lymphoid development which supports the observed phenotype of HRS cells of compromised B-cell differentiation.

SIX genes belong to the SINE class of homeobox genes numbering six members in human defining three subgroups: SIX1/2, SIX3/6, and SIX4/5 [1, 2]. The class-specific SIX-domain is located N-terminal of the homeodomain which belongs to the K50 class - both domains implementing protein-protein interactions [2, 33]. SIX genes regulate proliferation and differentiation and are part of a conserved GRN mediating development of the retina in addition to other placodal progenies [19, 20, 33]. Mutations in SIX genes have been detected in genetic disorders showing developmental defects in eyes, pituitary, ear and brain. Moreover, SIX genes are deregulated in several types of malignancies, including breast cancer and hepatocellular carcinoma (SIX1), lung cancer (SIX2), chondrosarcoma (SIX3), and acute T-cell leukemia (SIX6) [2]. Here, we have shown that SIX1 is aberrantly activated in HL and supports lymphomagenesis via deregulation of developmental genes.

The SIX1 locus at chromosome 14q23 demonstrated genomic gains in HL cells, indicating an activating role for aberrant SIX1 expression. This chromosomal region is amplified in HL in addition to other B-cell malignancies including acute lymphoblastic leukemia and diffuse large B-cell lymphoma [34–36]. However, deregulated target genes have not been hitherto identified in these malignant entities. Copy number alterations have been described in HL for several loci which show corresponding transcriptional deregulation of putative target genes, including gains at 2p15 (REL), 9p24 (JAK2) and 14q22 (OTX2) and deletions at 16q12 (CYLD) and Xp21 (CYBB) [18, 37–41]. Therefore, copy number variations represent a characteristic chromosomal abnormality in HL which unlike other common lymphomas eschews recurrent chromosome translocations.

During development of the preplacodal region SIX1 is regulated by several pathways including WNT-signalling [19]. Our data show that this pathway also modulates SIX1 expression in HL. In the embryonal setting the WNT-pathway mediates inhibition of SIX1 expression, contrasting with its activatory role in HL. While several aberrantly activated signalling pathways have been reported in HL, the WNT pathway has only recently been implicated in HL [42,43]. Cell lines may serve as models to investigate its regulation and function in HL.

MEF2C encodes a MADS-box TF expressed in spleen, muscle and heart and is accordingly involved in B-cell differentiation [44–46]. Knockout experiments in mice have demonstrated basic functions of MEF2C in proliferation and survival during B-cell development [47]. Aberrant activation has been reported in acute T-cell leukemia, whereby MEF2C is directly regulated by an oncogenic homeodomain TF, NKX2–5, or targeted by genomic deletions of non-coding regulatory regions [48–50]. Thus, physiologically MEF2C mediates lymphoid development of the B-cell lineage. Target genes of MEF2C are variously activated or repressed - the transcriptional activity is regulated by particular posttranslational modifications. Phosphorylation by MAPK7/ERK5 inhibits while acetylation enhances the repressive activity of the MEF2C protein [24–26]. Our data show in HL reduced MEF2C expression in addition to enhanced expression of MAPK7 and reduced expression of HDAC9 which deacetylates MEF2C. These findings together with the results of pharmacological inhibition of MAPK7 and HDACs indicate that reduced suppressive activity of MEF2C in HL promote SIX1 transcription in this B-cell malignancy. Aberrantly expressed MAPK7 has been recently identified in primary HRS cells at the protein level where it may activate the clustered homeobox gene HOXB9 [51]. Of note, physiological expression of MEF2C is detectable in the spleen but not in the thymus [44]. In contrast, expression of murine SIX1 is detectable in the thymus but not in the spleen (Suppl. Fig. 2), showing inverse expression patters. These findings, together with the results generated in this study demonstrating inhibition of GATA3 by SIX1, support a physiological role for SIX1 in early T-cell development which is suppressed by MEF2C in the development of B-cells.

SIX1 regulates its target genes in concert with cofactors [2], namely EYA1 and TLE4 which respectively enable activation or inhibition of SIX1 target genes in embryonal placodal precursor cells [32]. Our data support this dualistic mode of gene regulation in HL as shown for MSX1 and SPIB. Of note, SIX1 and EYA1 are targets of the onco-fusiongene MLL-ENL in leukemia [52], highlighting their role in hematopoietic malignancies. However, no common MLL-fusion genes were detected in HL cell lines L-428, L-540 or U-HO1 (Suppl. Fig. 3), specifically excluding this mechanism of aberrant gene activation. Nevertheless, these SIX1-cofactor interactions are performed by the SIX-domain and may have therapeutic implications [33]. The approach to inhibit oncogenic protein-protein interactions by particular peptides or small molecules has been realized for BCL6 in diffuse large B-cell lymphoma and may be applicable to SIX1 as well, disturbing its oncogenic transcriptional activity [53, 54].

The embryonal neural plate border region generates the neural crest and placodes which subsequently form major parts of the developing head including the jaw and the sensory nerves and organs [20]. SIX1 represents a master regulator of the preplacodal region during embryogenesis [19, 20]. Additional genes involved in both the development of these structures and the pathogenesis of HL includes MSX1, OTX2, GATA3, and FOXC1 [7, 16–18, 23, 43, 55, 56]. Thus, we observe conspicuous coincidence of genes and GRNs between this particular embryonal region and lymphoid development/malignancies. Furthermore, the evolutionary emergence of these fundamental cranial structures roughly coincided with lymphocytes which represent the adaptive immune system [57–61]. This situation may have abetted the coevolution of developmental innovations by deployment of the same pre-existing GRNs, nowadays reflected by their identical usage of several TFs in physiological development. Additional TFs manifesting this striking coincidence have been identified and are subject to ongoing research in our lab.

In this study, we identified SIX1 as a novel oncogene in HL disturbing B-cell differentiation by deregulation of basic developmental TF genes. We also highlight a role for SIX1 in early T-cell development via regulation of GATA3. Furthermore, our results reveal similarities of GRNs mediating T/B-cell lymphopoiesis, lymphomagenesis and the embryonal development of the neural plate border region, indicating cooption of genes and their regulatory interactions in both settings.

## MATERIALS AND METHODS

### Expression profiling

The GEO datasets GSE12453 [22] and GSE39134 [23] were used for in silico expression analysis of primary microdissected HRS cells obtained from HL patients and healthy donors. They were generated using the HG U133 Plus 2.0 gene chip platform from Affymetrix. Supplemented online tools at the National Center for Biotechnology Information were used for data analysis [62]. Additionally, we used R-based tools to calculate correlations statistically and to illustrate the data by boxplots (http://cran.r-project.org/).

Gene expression microarray profiling data of cell lines were obtained using the HG U133 Plus 2.0 gene chip (Affymetrix, High Wycombe, UK). Datasets for HL cell lines HDLM-2, KM-H2, L428, L-540, L-1236 and SUP-HD1 were generated by Prof. Andreas Rosenwald (Institute of Pathology, University of Würzburg, Germany) as described previously [51]. Datasets for HL cell line U-HO1 were generated by Dr. Robert Geffers (Genome Analytics Facility, Helmholtz Centre for Infection Research, Braunschweig, Germany). Data for all 7 HL cell lines are publicly available in the supplement of a recent publication [18]. Data analysis was performed using Microsoft Excel. For creation of heat maps we used CLUSTER version 2.11 and TREEVIEW version 1.60 (http://rana.lbl.gov/EisenSoftware.htm).

### Cell lines and treatments

HL cell lines are held by the DSMZ (Braunschweig, Germany) and were cultivated as described previously [63]. Treatments of cell lines were performed for 20 h with 10 μg/ml Trichostatin A (TSA) (Sigma, Taufkirchen, Germany), 10 μM XMD8–92 (Biozol, Eching, Germany), 10 μM IWR1 (R&D Systems, Wiesbaden, Germany), 20 ng/ml recombinant human Bone Morphogenic Protein 4 (BMP4) protein, 20 ng/ml Fibroblast Growth Factor 2 (FGF2) protein, 20 ng/ml Transforming Growth Factor beta (TGFb), or 20 ng/ml WNT5B protein (R&D Systems). Gene specific siRNA oligonucleotides and AllStars negative Control siRNA (siControl) were obtained from Qiagen (Hilden, Germany). Expression constructs for MEF2C and SIX1 were cloned into the vector pCMV6-XL5 and obtained from Origene (Wiesbaden, Germany). SiRNAs (80 pmol), expression constructs (2 μg), reporter constructs (1 μg), and luciferase reporter construct (200 ng) were transfected into 1×10^6^ cells by electroporation using the EPI-2500 impulse generator (Fischer, Heidelberg, Germany) at 350 V for 10 ms. Treated cells were subsequently harvested after 20 h.

### Polymerase chain-reaction (PCR) analyses

Total RNA was extracted using TRIzol reagent (Invitrogen, Darmstadt, Germany). Primary human material used in this study was commercially obtained - total human RNA isolated from peripheral blood mononuclear cells (PBC), thymus, lymph node (LN), and bone marrow (BM) from Clontech (Saint-Germain-en-Laye, France), and RNA from peripheral CD19-positive B-cells and CD3-positive T-cells from Miltenyi Biotec (Bergisch Gladbach, Germany). cDNA was synthesized from 5 μg RNA by random priming using Superscript II (Invitrogen).

Qualitative reverse transcription (RT) PCR was performed using taqpol (Qiagen), oligonucleotides as described elsewhere [64], and the thermocycler TGradient (Biometra, Göttingen, Germany). Amplification of ETV6 served as positive control.

Real-time quantitative gene expression analysis (RQ-PCR) was performed with the 7500 Real-time System, using commercial buffer and primer sets (Applied Biosystems, Darmstadt, Germany). Quantification of MSX1 was performed as described recently [7]. For normalization of expression levels we analyzed the transcript of TATA box binding protein (TBP). Quantitative analyses were performed in triplicate. Preparation of genomic DNA was performed using the High Pure PCR Template Preparation Kit (Roche Diagnostics, Mannheim, Germany). For genomic copy number quantification we used the following oligonucleotides hybridizing within the coding region of the SIX1 gene: forward 5′-TTACGCAGGAGCAAGTGGCG-3′, reverse 5′-CGCTCTCGTTCTTGTGCAGG-3′. As reference we used the MEF2C gene as reported previously [49]. Standard deviations are presented in the figures as error bars. The statistical significance was assessed by Student's T-Test and the calculated *p*-values are indicated by asterisks (*p < 0.05, **p < 0.01, ***p < 0.001, n.s. no significance).

### Protein analyses

Western blots were generated by the semi-dry method. Proteins obtained from cell line lysates using SIGMAFast protease inhibitor cocktail (Sigma) were transferred onto nitrocellulose membranes (Bio-Rad, München, Germany) and blocked with 5% dry milk powder dissolved in phosphate-buffered-saline buffer (PBS). The following antibodies were used: alpha-Tubulin (Sigma), and SIX1 (Novus Biologicals Europe, Cambridge, UK). For loading control the blots were stained with Poinceau (Sigma) and then detection of alpha-Tubulin (TUBA) was performed. Secondary antibodies were linked to peroxidase for detection by Western-Lightning-ECL (Perkin Elmer, Waltham, MA, USA). Documentation was performed using the digital system ChemoStar Imager (INTAS, Göttingen, Germany).

### Chromosomal analysis

Chromosomal analysis by fluorescent in-situ hybridization (FISH) was performed as described previously [65]. RP11-BAC clones were obtained from BacPac Resources, Children's Hospital Oakland Research Institute (CA, USA), insert DNA harvested using the Big BAC DNA Kit (Princeton Separations, Adelphia, NJ, USA) and directly labelled by nick translation with dUTP-fluors (Dyomics, Jena, Germany). Whole chromosome paint (WCP) probes were obtained from Applied Spectral Imaging (Neckarhausen, Germany). Fluorescent images were captured and analyzed with an Axio-Imager microscope (Zeiss, Göttingen, Germany) configured to a dual Spectral Imaging FISH system (Applied Spectral Imaging).

### Reporter gene assay

For creation of reporter gene constructs we combined a reporter with regulatory genomic fragments derived from the upstream regions of SIX1 and SPIB. We cloned genomic PCR products of the corresponding upstream regions (regulator) and of the HOXA9 gene, comprising exon1-intron1-exon2 (reporter), into the *Hind*III/*Bam*HI and *Eco*RI sites, respectively, of the expression vector pcDNA3 downstream of the CMV enhancer [7]. The oligonucleotides used for the amplification of the SIX1-regulator were obtained from MWG Eurofins (Ebersberg, Germany) and the sequences were as follows: forward 5′-AGAAGCTTGGGAGAAGTTTCAAATTACTAGG-3′, reverse 5′-ATGGATCCGAGGCCCAGTGACAAGGA AAGC-3′. The oligonucleotides used for the amplification of the SPIB-regulator were as follows: forward 5′-GGAAGCTTCTGGAAGACCAGGAGCAGCTC-3′, reverse 5′-GCCCGCTGCAGCCGCCAACC-3′. Constructs were validated by sequence analysis (MWG Eurofins). Commercial HOXA9 and TBP assays were used for RQ-PCR to quantify the spliced reporter-transcript, corresponding to the regulator activity. A cotransfected commercial luciferase construct served as transfection control and was quantified by the Luciferase Assay System (Promega, Mannheim, Germany) using the luminometer Lumat LB9501 (Berthold Technologies, Bad Wildbad, Germany).

## SUPPLEMENTARY FIGURES AND TABLES







## References

[1] Holland PW, Booth HA, Bruford EA (2007). Classification and nomenclature of all human homeobox genes. BMC Biol.

[2] Christensen KL, Patrick AN, McCoy EL, Ford HL (2008). The Six family of homeobox genes in development and cancer. Adv Cancer Res.

[3] Abate-Shen C (2002). Deregulated homeobox gene expression in cancer: cause or consequence?. Nat Rev Cancer.

[4] Argiropoulos B, Humphries RK (2007). Hox genes in hematopoiesis and leukemogenesis. Oncogene.

[5] Nagel S, Ehrentraut S, Meyer C, Kaufmann M, Drexler HG, MacLeod RA (2015). Repressed BMP signaling reactivates NKL homeobox gene MSX1 in a T-ALL subset. Leuk Lymphoma.

[6] Nagel S, Ehrentraut S, Meyer C, Kaufmann M, Drexler HG, MacLeod RA (2014). Oncogenic deregulation of NKL homeobox gene MSX1 in mantle cell lymphoma. Leuk Lymphoma.

[7] Nagel S, Schneider B, Meyer C, Kaufmann M, Drexler HG, Macleod RA (2012). Transcriptional deregulation of homeobox gene ZHX2 in Hodgkin lymphoma. Leuk Res.

[8] Jojic V, Shay T, Sylvia K, Zuk O, Sun X, Kang J, Regev A, Koller D, Best AJ, Knell J, Goldrath A, Joic V, Koller D, Shay T, Regev A, Cohen N, Brennan P, Brenner M, Kim F, Rao TN, Wagers A, Heng T, Ericson J, Rothamel K, Ortiz-Lopez A, Mathis D, Benoist C, Bezman NA, Sun JC, Min-Oo G, Kim CC, Lanier LL, Miller J, Brown B, Merad M, Gautier EL, Jakubzick C, Randolph GJ, Monach P, Blair DA, Dustin ML, Shinton SA, Hardy RR, Laidlaw D, Collins J, Gazit R, Rossi DJ, Malhotra N, Sylvia K, Kang J, Kreslavsky T, Fletcher A, Elpek K, Bellemarte-Pelletier A, Malhotra D, Turley S, Immunological Genome Project Consortium (2013). Identification of transcriptional regulators in the mouse immune system. Nat Immunol.

[9] Nagel S, Venturini L, Przybylski GK, Grabarczyk P, Meyer C, Kaufmann M, Battmer K, Schmidt CA, Drexler HG, Scherr M, Macleod RA (2009). NK-like homeodomain proteins activate NOTCH3-signaling in leukemic T-cells. BMC Cancer.

[10] Medvedovic J, Ebert A, Tagoh H, Busslinger M (2011). Pax5: a master regulator of B cell development and leukemogenesis. Adv Immunol.

[11] Hystad ME, Myklebust JH, Bø TH, Sivertsen EA, Rian E, Forfang L, Munthe E, Rosenwald A, Chiorazzi M, Jonassen I, Staudt LM, Smeland EB (2007). Characterization of early stages of human B cell development by gene expression profiling. J Immunol.

[12] Küppers R (2009). The biology of Hodgkin's lymphoma. Nat Rev Cancer.

[13] Hertel CB, Zhou XG, Hamilton-Dutoit SJ, Junker S (2002). Loss of B cell identity correlates with loss of B cell-specific transcription factors in Hodgkin/Reed-Sternberg cells of classical Hodgkin lymphoma. Oncogene.

[14] Küppers R, Klein U, Schwering I, Distler V, Bräuninger A, Cattoretti G, Tu Y, Stolovitzky GA, Califano A, Hansmann ML, Dalla-Favera R (2003). Identification of Hodgkin and Reed-Sternberg cell-specific genes by gene expression profiling. J Clin Invest.

[15] Schwering I, Bräuninger A, Klein U, Jungnickel B, Tinguely M, Diehl V, Hansmann ML, Dalla-Favera R, Rajewsky K, Küppers R (2003). Loss of the B-lineage-specific gene expression program in Hodgkin and Reed-Sternberg cells of Hodgkin lymphoma. Blood.

[16] Mathas S, Janz M, Hummel F, Hummel M, Wollert-Wulf B, Lusatis S, Anagnostopoulos I, Lietz A, Sigvardsson M, Jundt F, Jöhrens K, Bommert K, Stein H, Dörken B (2006). Intrinsic inhibition of transcription factor E2A by HLH proteins ABF-1 and Id2 mediates reprogramming of neoplastic B cells in Hodgkin lymphoma. Nat Immunol.

[17] Atayar C, Poppema S, Blokzijl T, Harms G, Boot M, van den Berg A (2005). Expression of the T-cell transcription factors, GATA-3 and T-bet, in the neoplastic cells of Hodgkin lymphomas. Am J Pathol.

[18] Nagel S, Ehrentraut S, Meyer C, Kaufmann M, Drexler HG, MacLeod RA (2015). Aberrantly expressed OTX homeobox genes deregulate B-cell differentiation in Hodgkin lymphoma. PLoS One.

[19] Schlosser G (2014). Early embryonic specification of vertebrate cranial placodes. Wiley Interdiscip Rev Dev Biol.

[20] Moody SA, LaMantia AS (2015). Transcriptional regulation of cranial sensory placode development. Curr Top Dev Biol.

[21] Gestri G, Carl M, Appolloni I, Wilson SW, Barsacchi G, Andreazzoli M (2005). Six3 functions in anterior neural plate specification by promoting cell proliferation and inhibiting Bmp4 expression. Development.

[22] Brune V, Tiacci E, Pfeil I, Döring C, Eckerle S, van Noesel CJ, Klapper W, Falini B, von Heydebreck A, Metzler D, Bräuninger A, Hansmann ML, Küppers R (2008). Origin and pathogenesis of nodular lymphocyte-predominant Hodgkin lymphoma as revealed by global gene expression analysis. J Exp Med.

[23] Steidl C, Diepstra A, Lee T, Chan FC, Farinha P, Tan K, Telenius A, Barclay L, Shah SP, Connors JM, van den Berg A, Gascoyne RD (2012). Gene expression profiling of microdissected Hodgkin Reed-Sternberg cells correlates with treatment outcome in classical Hodgkin lymphoma. Blood.

[24] Kato Y, Kravchenko VV, Tapping RI, Han J, Ulevitch RJ, Lee JD (1997). BMK1/ERK5 regulates serum-induced early gene expression through transcription factor MEF2C. EMBO J.

[25] Ma K, Chan JK, Zhu G, Wu Z (2005). Myocyte enhancer factor 2 acetylation by p300 enhances its DNA binding activity, transcriptional activity, and myogenic differentiation. Mol Cell Biol.

[26] Grégoire S, Yang XJ (2005). Association with class IIa histone deacetylases upregulates the sumoylation of MEF2 transcription factors. Mol Cell Biol.

[27] Brooke NM, Holland PW (2003). The evolution of multicellularity and early animal genomes. Curr Opin Genet Dev.

[28] De Robertis EM (2008). Evo-devo: variations on ancestral themes. Cell.

[29] Tsai FY, Keller G, Kuo FC, Weiss M, Chen J, Rosenblatt M, Alt FW, Orkin SH (1994). An early haematopoietic defect in mice lacking the transcription factor GATA-2. Nature.

[30] Schneider EM, Torlakovic E, Stühler A, Diehl V, Tesch H, Giebel B (2004). The early transcription factor GATA-2 is expressed in classical Hodgkin's lymphoma. J Pathol.

[31] Rothenberg EV, Scripture-Adams DD (2008). Competition and collaboration: GATA-3, PU.1, and Notch signaling in early T-cell fate determination. Semin Immunol.

[32] Brugmann SA, Pandur PD, Kenyon KL, Pignoni F, Moody SA (2004). Six1 promotes a placodal fate within the lateral neurogenic ectoderm by functioning as both a transcriptional activator and repressor. Development.

[33] Kawakami K, Sato S, Ozaki H, Ikeda K (2000). Six family genes - structure and function as transcription factors and their roles in development. Bioessays.

[34] Steidl C, Telenius A, Shah SP, Farinha P, Barclay L, Boyle M, Connors JM, Horsman DE, Gascoyne RD (2010). Genome-wide copy number analysis of Hodgkin Reed-Sternberg cells identifies recurrent imbalances with correlations to treatment outcome. Blood.

[35] Gruszka-Westwood AM, Horsley SW, Martinez-Ramirez A, Harrison CJ, Kempski H, Moorman AV, Ross FM, Griffiths M, Greaves MF, Kearney L (2004). Comparative expressed sequence hybridization studies of high-hyperdiploid childhood acute lymphoblastic leukemia. Genes Chromosomes Cancer.

[36] Guo Y, Takeuchi I, Karnan S, Miyata T, Ohshima K, Seto M (2014). Array-comparative genomic hybridization profiling of immunohistochemical subgroups of diffuse large B-cell lymphoma shows distinct genomic alterations. Cancer Sci.

[37] Joos S, Küpper M, Ohl S, von Bonin F, Mechtersheimer G, Bentz M, Marynen P, Möller P, Pfreundschuh M, Trümper L, Lichter P (2000). Genomic imbalances including amplification of the tyrosine kinase gene JAK2 in CD30+ Hodgkin cells. Cancer Res.

[38] Joos S, Menz CK, Wrobel G, Siebert R, Gesk S, Ohl S, Mechtersheimer G, Trümper L, Möller P, Lichter P, Barth TF (2002). Classical Hodgkin lymphoma is characterized by recurrent copy number gains of the short arm of chromosome 2. Blood.

[39] Martín-Subero JI, Gesk S, Harder L, Sonoki T, Tucker PW, Schlegelberger B, Grote W, Novo FJ, Calasanz MJ, Hansmann ML, Dyer MJ, Siebert R (2002). Recurrent involvement of the REL and BCL11A loci in classical Hodgkin lymphoma. Blood.

[40] Giefing M, Winoto-Morbach S, Sosna J, Döring C, Klapper W, Küppers R, Böttcher S, Adam D, Siebert R, Schütze S (2013). Hodgkin-Reed-Sternberg cells in classical Hodgkin lymphoma show alterations of genes encoding the NADPH oxidase complex and impaired reactive oxygen species synthesis capacity. PLoS One.

[41] Schmidt A, Schmitz R, Giefing M, Martin-Subero JI, Gesk S, Vater I, Massow A, Maggio E, Schneider M, Hansmann ML, Siebert R, Küppers R (2010). Rare occurrence of biallelic CYLD gene mutations in classical Hodgkin Zlymphoma. Genes Chromosomes Cancer.

[42] Sohlbach K, Moll R, Goßmann J, Nowak O, Barth P, Neubauer A, Huynh MQ (2012). β-Catenin signaling: no relevance in Hodgkin lymphoma?. Leuk Lymphoma.

[43] Nagel S, Meyer C, Kaufmann M, Drexler HG, MacLeod RA (2014). Deregulated FOX genes in Hodgkin lymphoma. Genes Chromosomes Cancer.

[44] Martin JF, Schwarz JJ, Olson EN (1993). Myocyte enhancer factor (MEF) 2C: a tissue-restricted member of the MEF-2 family of transcription factors. Proc Natl Acad Sci USA.

[45] Swanson BJ, Jäck HM, Lyons GE (1998). Characterization of myocyte enhancer factor 2 (MEF2) expression in B and T cells: MEF2C is a B cell-restricted transcription factor in lymphocytes. Mol Immunol.

[46] Debnath I, Roundy KM, Pioli PD, Weis JJ, Weis JH (2013). Bone marrow-induced Mef2c deficiency delays B-cell development and alters the expression of key B-cell regulatory proteins. Int Immunol.

[47] Wilker PR, Kohyama M, Sandau MM, Albring JC, Nakagawa O, Schwarz JJ, Murphy KM (2008). Transcription factor Mef2c is required for B cell proliferation and survival after antigen receptor stimulation. Nat Immunol.

[48] Nagel S, Meyer C, Quentmeier H, Kaufmann M, Drexler HG, MacLeod RA (2008). MEF2C is activated by multiple mechanisms in a subset of T-acute lymphoblastic leukemia cell lines. Leukemia.

[49] Nagel S, Venturini L, Meyer C, Kaufmann M, Scherr M, Drexler HG, Macleod RA (2011). Transcriptional deregulation of oncogenic myocyte enhancer factor 2C in T-cell acute lymphoblastic leukemia. Leuk Lymphoma.

[50] Homminga I, Pieters R, Langerak AW, de Rooi JJ, Stubbs A, Verstegen M, Vuerhard M, Buijs-Gladdines J, Kooi C, Klous P, van Vlierberghe P, Ferrando AA, Cayuela JM, Verhaaf B, Beverloo HB, Horstmann M, de Haas V, Wiekmeijer AS, Pike-Overzet K, Staal FJ, de Laat W, Soulier J, Sigaux F, Meijerink JP (2011). Integrated transcript and genome analyses reveal NKX2–1 and MEF2C as potential oncogenes in T cell acute lymphoblastic leukemia. Cancer Cell.

[51] Nagel S, Burek C, Venturini L, Scherr M, Quentmeier H, Meyer C, Rosenwald A, Drexler HG, MacLeod RA (2007). Comprehensive analysis of homeobox genes in Hodgkin lymphoma cell lines identifies dysregulated expression of HOXB9 mediated via ERK5 signaling and BMI1. Blood.

[52] Wang QF, Wu G, Mi S, He F, Wu J, Dong J, Luo RT, Mattison R, Kaberlein JJ, Prabhakar S, Ji H, Thirman MJ (2011). MLL fusion proteins preferentially regulate a subset of wild-type MLL target genes in the leukemic genome. Blood.

[53] Polo JM, Dell'Oso T, Ranuncolo SM, Cerchietti L, Beck D, Da Silva GF, Prive GG, Licht JD, Melnick A (2004). Specific peptide interference reveals BCL6 transcriptional and oncogenic mechanisms in B-cell lymphoma cells. Nat Med.

[54] Cerchietti LC, Ghetu AF, Zhu X, Da Silva GF, Zhong S, Matthews M, Bunting KL, Polo JM, Farès C, Arrowsmith CH, Yang SN, Garcia M, Coop A, Mackerell AD, Privé GG, Melnick A (2010). A small-molecule inhibitor of BCL6 kills DLBCL cells *in vitro* and *in vivo*. Cancer Cell.

[55] Schlosser G, Ahrens K (2004). Molecular anatomy of placode development in Xenopus laevis. Dev Biol.

[56] Nelms BL, Labosky PA (2010). Transcriptional Control of Neural Crest Development.

[57] Tomsa JM, Langeland JA (1999). Otx expression during lamprey embryogenesis provides insights into the evolution of the vertebrate head and jaw. Dev Biol.

[58] Chen JY (2009). The sudden appearance of diverse animal body plans during the Cambrian explosion. Int J Dev Biol.

[59] Pancer Z, Cooper MD (2006). The evolution of adaptive immunity. Annu Rev Immunol.

[60] Boehm T, McCurley N, Sutoh Y, Schorpp M, Kasahara M, Cooper MD (2012). VLR-based adaptive immunity. Annu Rev Immunol.

[61] Butler AB (2000). Chordate evolution and the origin of craniates: an old brain in a new head. Anat Rec.

[62] Huang da W, Sherman BT, Lempicki (2009). Systematic and integrative analysis of large gene lists using DAVID bioinformatics resources. Nat Protoc.

[63] Drexler HG (2010). Guide to Leukemia-Lymphoma Cell Lines.

[64] van Dongen JJ, Macintyre EA, Gabert JA, Delabesse E, Rossi V, Saglio G, Gottardi E, Rambaldi A, Dotti G, Griesinger F, Parreira A, Gameiro P, Diáz MG, Malec M, Langerak AW, San Miguel JF, Biondi A (1999). Standardized RT-PCR analysis of fusion gene transcripts from chromosome aberrations in acute leukemia for detection of minimal residual disease. Report of the BIOMED-1 Concerted Action: investigation of minimal residual disease in acute leukemia. Leukemia.

[65] Macleod RA, Kaufmann M, Drexler HG (2011). Cytogenetic analysis of cancer cell lines. Methods Mol Biol.

