# Supplementary material for: Aberrant expression of homeobox gene SIX1 in Hodgkin lymphoma
Source: Oncotarget. 2015 Oct 12;6(37):40112–26. doi: 10.18632/oncotarget.5556 (PMC4741883; doi:10.18632/oncotarget.5556)
Supplement: Supplementary file 1 [file oncotarget-06-40112-s001.pdf]

## SUPPLEMENTARY FIGURES AND TABLE

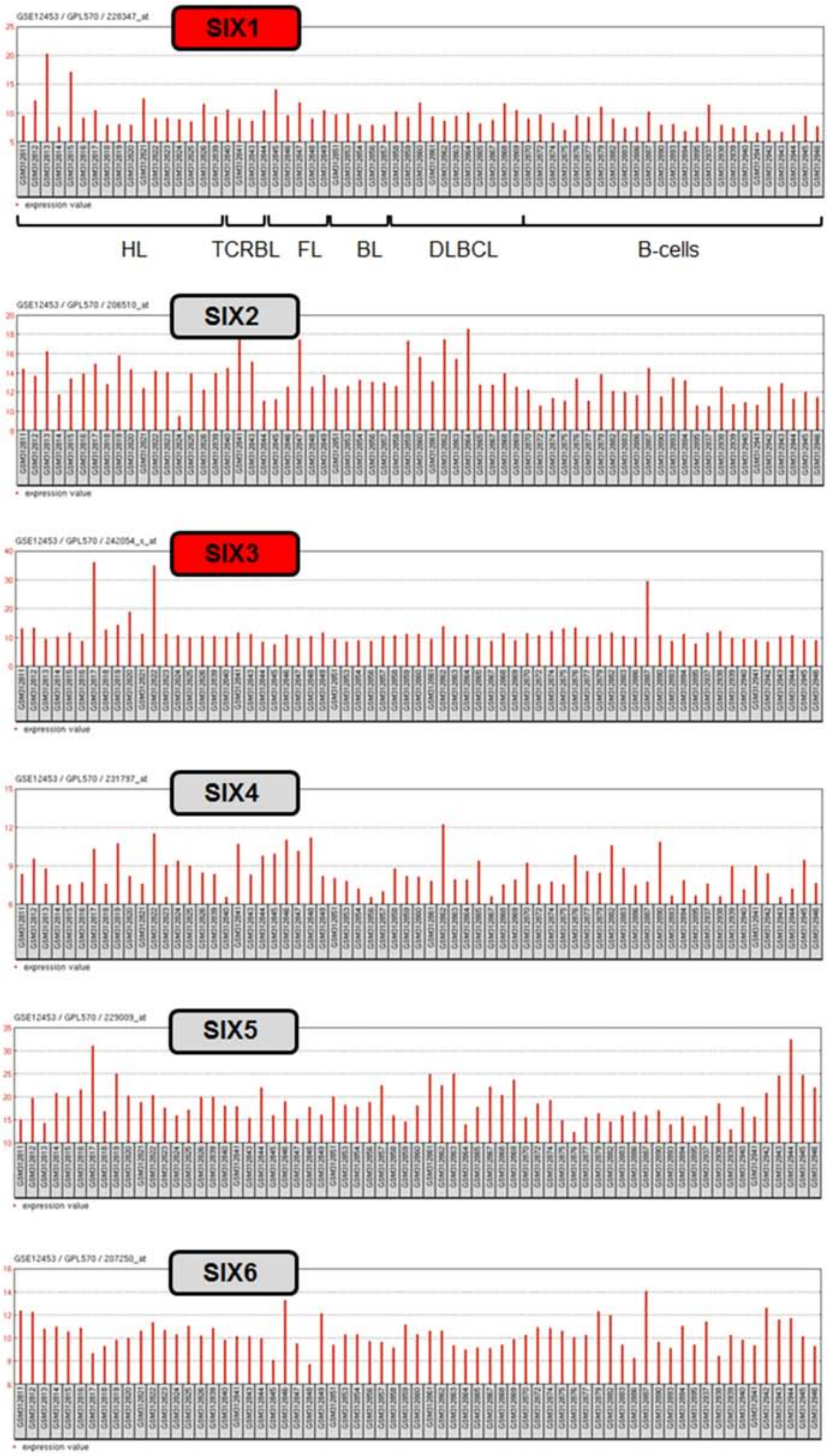


**Supplementary Figure S1: SIX gene expression in HL.** Profiling data of SIX-gene expression levels in HL patients for data set GSE12453 **A**. and data set GSE39134.

(*Continued*)

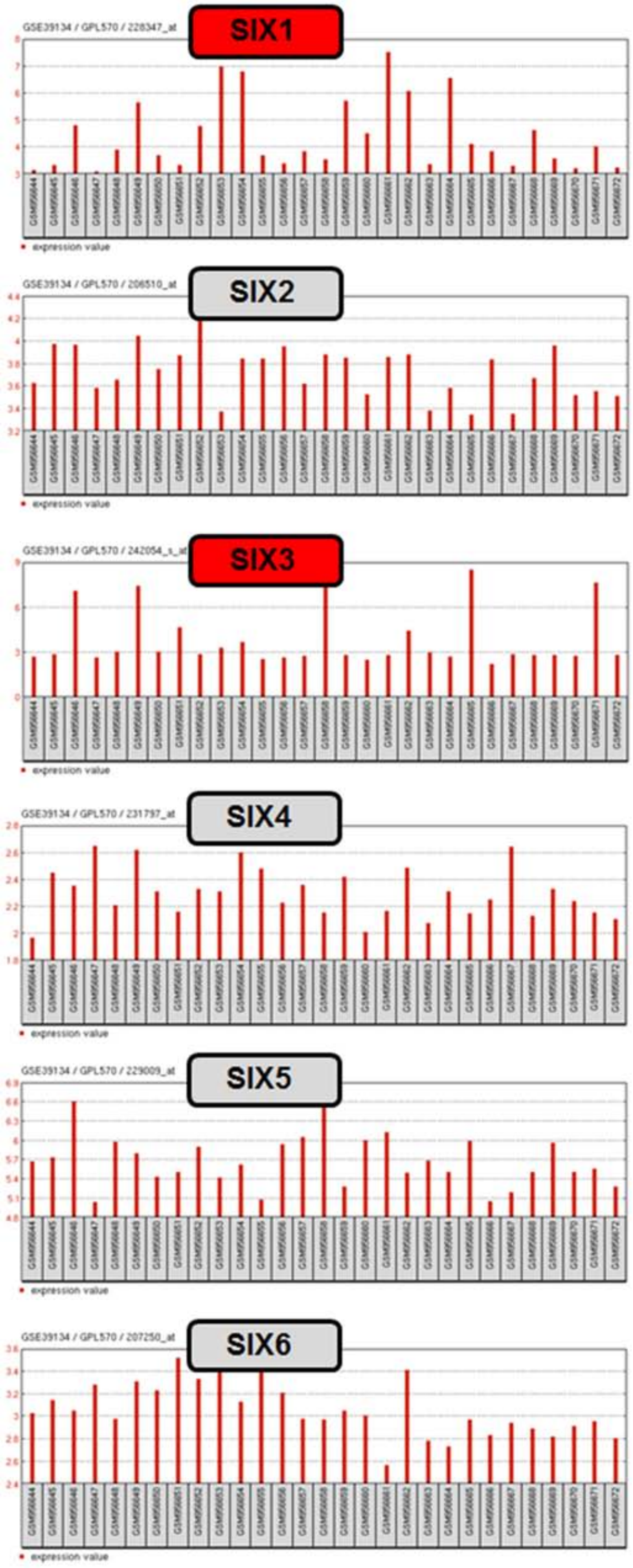


**Supplementary Figure S1 (*Continued*): B.** Note that in both data sets SIX1 and SIX3 show conspicuously enhanced expression levels in subsets of HL patients.

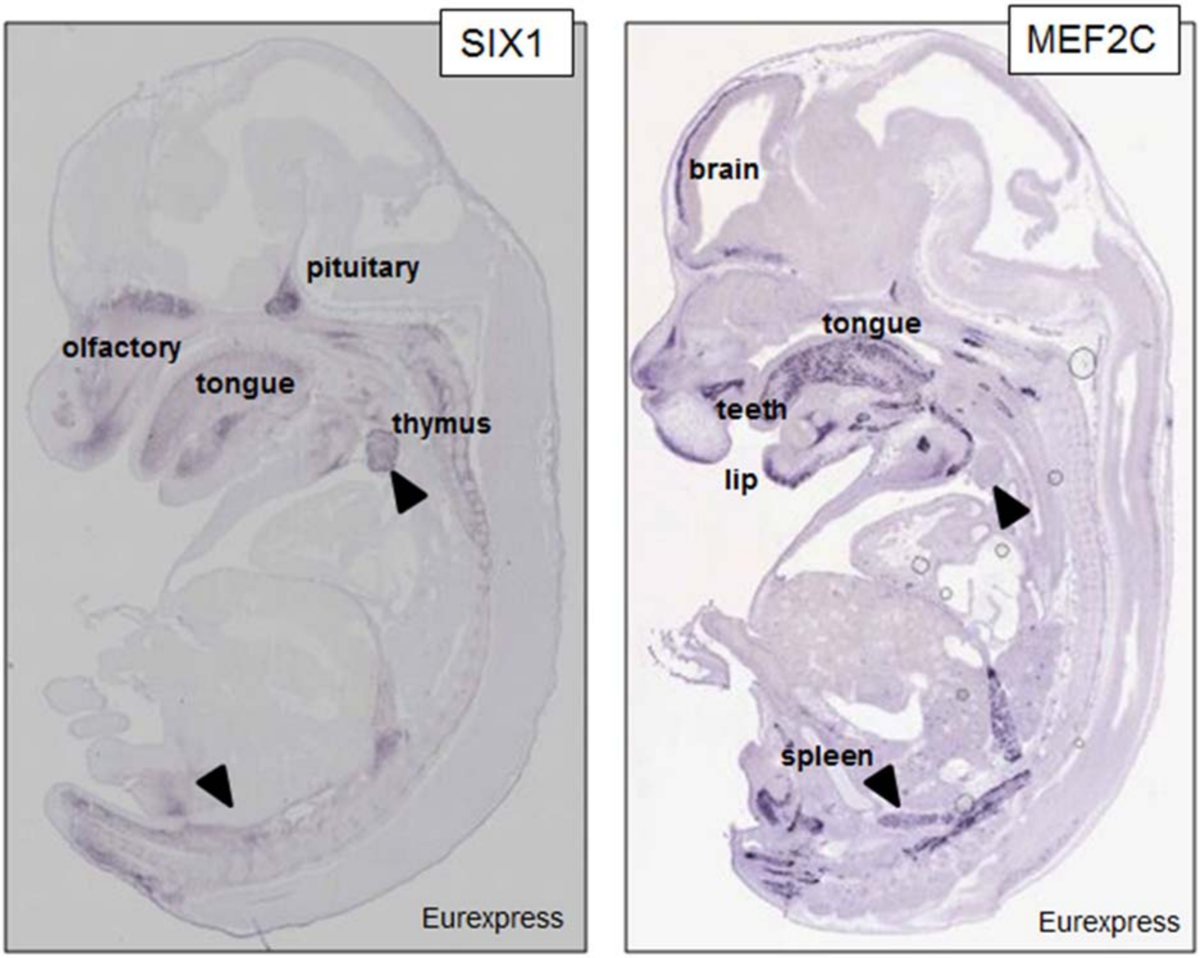


**Supplementary Figure S2: Embryonal expression of SIX1 and MEF2C.** In situ hybridization results showing SIX1 and MEF2C expression in mouse embryos using in silico data obtained from the Transcriptome Atlas Database for Mouse Embryo (Eurexpress). Note SIX1 is expressed in placodal tissues (pituitary and olfactory) and in the thymus, supporting a regulatory role in T-cell development. In contrast, MEF2C is detectable in the spleen but not in the thymus.

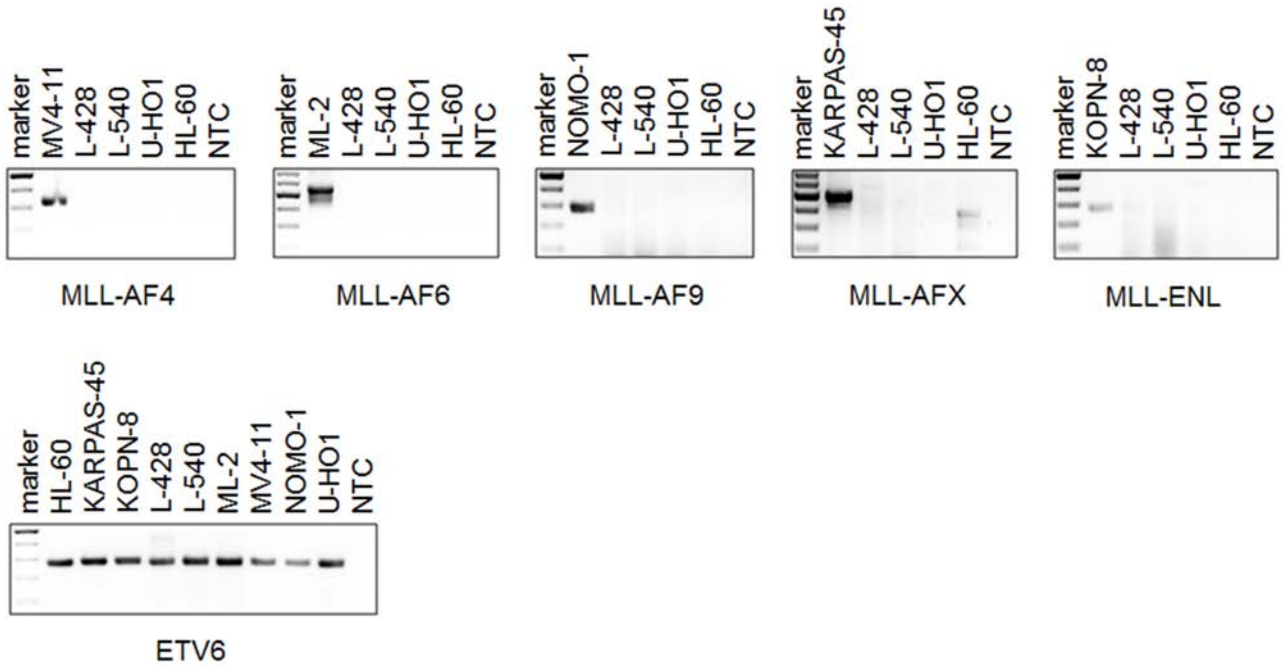


**Supplementary Figure S3: MLL fusion-gene analysis in HL cell lines.** RT-PCR analysis of 5 frequent MLL fusion genes in HL cell lines and control cell lines: HL-60 negative control, MV-4-11 positive control for MLL-AF4, ML-2 for MLL-AF6, NOMO-1 for MLL-AF9, KARPAS-45 for MLL-AFX, and KOPN-8 for MLL-ENL. ETV6 served as control for cDNA integrity, NTC: no template control.

## Supplementary Table S1: Expression profiling data of 7 HL cell lines

The data were grouped according to SIX1-positive cell lines (L-428, L-540, U-HO1) and SIX1-negative cell lines (HDLM-2, KM-H2, SUP-HD1). The data are ordered according to elevated expression levels (A) and reduced expression levels (B) in SIX1-positive cell lines, indicating genes correlated with SIX1.
